# The Clinical Significance of Soluble Programmed Cell Death-Ligand 1 (sPD-L1) in Patients With Gliomas

**DOI:** 10.3389/fonc.2020.00009

**Published:** 2020-01-23

**Authors:** Shujun Liu, Yadi Zhu, Chenxi Zhang, Xiangrui Meng, Bo Sun, Guojun Zhang, Yubo Fan, Xixiong Kang

**Affiliations:** ^1^Laboratory Diagnosis Center, Beijing Tiantan Hospital, Capital Medical University, Beijing, China; ^2^Beijing Engineering Research Center of Immunological Reagents and Clinical Research, Beijing, China; ^3^Beijing Advanced Innovation Center for Biomedical Engineering, Beihang University, Beijing, China

**Keywords:** glioma, soluble programmed cell death-ligand 1, biomarker, cerebrospinal fluid, inflammatory markers

## Abstract

**Background:** Soluble PD-L1 (sPD-L1) in the circulation has been documented to activate global immunosuppression and is considered a predictor of negative clinical outcomes in several malignances. However, the clinical significance of sPD-L1 in the peripheral blood and cerebrospinal fluid (CSF) of patients with glioma remains unclear.

**Objective:** The aim of this study was to detect the correlations of sPD-L1 with clinical features in brain tumors and assess the diagnostic value of this protein in gliomas.

**Methods:** Serum samples were obtained from 73 patients with glioma, 20 patients with meningioma, and 49 healthy controls (HCs) in this study. In total, 31 CSF samples were collected from the matched glioma patients, and seven samples were collected from the matched meningioma patients. The expression of serum sPD-L1 in the glioma cohort was followed for 20 days after surgery to examine the kinetics in the circulation. Inflammatory markers were evaluated based on preoperative blood parameters. The sPD-L1 levels in the serum and CSF were determined by enzyme-linked immunosorbent assay (ELISA). The logistic regression model was used to assess the independent associations of sPD-L1 with gliomas, including high-grade gliomas.

**Results:** Serum and CSF levels of sPD-L1 were significantly elevated in patients with gliomas compared to those with meningiomas and HCs. Additionally, increased levels of sPD-L1 were observed in relatively advanced tumors. sPD-L1 overexpression in the CSF appears to be more representative of aggressive tumor features than overexpression in the serum. For glioma diagnosis, both serum and CSF sPD-L1 showed significant value in the diagnosis and stratification of glioma, and the best diagnostic performance was obtained with serum sPD-L1 rather than blood-based inflammatory markers. In addition, a descending trend in the level of serum sPD-L1 was observed in postoperative patients.

**Conclusion:** In gliomas, elevated circulating and CSF sPD-L1 levels are associated with aggressive biological activities. The results of the current study suggest that sPD-L1 is a promising biomarker for gliomas that can be used in clinical practice.

## Introduction

Gliomas, which account for the majority of central nervous system (CNS) tumors, are correlated with high rates of recurrence and mortality (1). However, molecular information such as the mutational status of isocitrate dehydrogenase (IDH) genes and the combined deletion of chromosome arms 1p and 19q (1p/19q codeletion) is integral to the 2016 World Health Organization (WHO) criteria for gliomas (2). Thus, these updates have shifted treatment approaches from histological type-based therapy to genotype-based therapy (3). Despite some advances in neurosurgical resection and treatment regimens, the prognosis of patients with high-grade gliomas remains dismal (4). The current methods for diagnosing and monitoring gliomas are seriously dependent on invasive procedures such as biopsy or surgery, which create challenges in the management of cancer patients (5). Accordingly, identifying a reliable liquid-based biomarker that can be measured quickly and safely is an attractive clinical approach.

For liquid-based biomarkers, several blood-based inflammatory markers, such as the neutrophil-to-lymphocyte ratio (NLR), derived NLR (dNLR), and platelet-to-lymphocyte ratio (PLR), have been highlighted for their pivotal roles in the stratification and prognosis of gliomas (6, 7). Likewise, hematological biomarkers used for indicating nutritional and immunological statuses, such as the prognostic nutrition index (PNI) and albumin-to-globulin ratio (AGR), have been identified as predictive markers in advanced brain tumors (8, 9). In contrast, other reports concluded that these markers were prone to various biases (10).

Recently, the discovery of immune checkpoint molecules, such as programmed death-1 (PD-1) protein and its ligand, namely, programmed death-ligand 1 (PD-L1), has provided novel therapeutic targets. Considering the lack of effective treatments for gliomas, immunotherapy, especially anti-PD-1/PD-L1 antibodies, has brought hope for brain malignances (11, 12). In fact, PD-L1 expression on the surface of tumor cells, one of the potential indicators for checkpoint inhibitor use (13), was found to be detectable in a portion of glioma patients (14, 15). However, ample tumor tissue is still required for assessing PD-L1 expression in tumor cells and the tumor microenvironment (16). Appealingly, PD-L1 is expressed not only on the surface of cells but also in a soluble form in the circulation, which is thought to be released from PD-L1-positive cells (17).

Similar to membrane-binding PD-L1, soluble PD-L1 (sPD-L1) is currently thought to contribute to systemic immunosuppression (18, 19). Recently, a few published reports have shown that sPD-L1 is an indispensable predictor in various types of cancer (20–22), especially for checkpoint blockade treatments (23, 24). In addition, some studies have referred to circulating sPD-L1 as a surrogate marker for tumor PD-L1 expression (25). Given that both sPD-L1 and the previously mentioned blood markers have been regarded as indicators of the host immune state (21), we were interested in determining the associations between sPD-L1 measurements and peripheral blood markers in gliomas.

It is worth mentioning that our previous study showed that sPD-L1 in the peripheral blood was a potential predictor for the diagnosis and prognosis of preoperative patients with glioma. Nevertheless, blood is one of the easiest biofluids to obtain, but it is not the optimal fluid for collecting precise biomarkers derived from brain tumors due to the isolation established by the blood-brain barrier (BBB) (26). Instead, the cerebrospinal fluid (CSF) directly contacts the interstitial fluid in the CNS; thus, the CSF is considered to be the optimal source of markers for CNS tumors (27). It is still unclear whether the circulating sPD-L1 level can reflect the expression level in the CSF because insufficient findings on central and peripheral sPD-L1 measurements in gliomas have been reported.

As such, in this study, we aimed to elucidate these issues through the following clinical evaluations: (a) exploring the relationships of serum sPD-L1 with blood-based inflammatory markers; (b) detecting the concentration of sPD-L1 in the CSF and illustrating the relationship between sPD-L1 measurements in the serum and CSF; (c) determining the correlations of sPD-L1 levels with glioma classification, histological characteristics and molecular features; and (d) evaluating the predictive significance of sPD-L1 in gliomas. This report may provide new clues for developing and validating reliable and minimally invasive biomarkers for CNS tumors in future clinical practices.

## Methods

The study protocol was approved by the ethics committee of Beijing Tiantan Hospital.

### Study Subjects

Patients with brain tumors who underwent magnetic resonance imaging (MRI) between January and April 2019 were consecutively followed. In total, 73 patients with histologically confirmed glioma were included in this study. Furthermore, 20 patients with meningioma were sex-matched with the glioma patients and enrolled in this study. All patients underwent maximal safe surgical resection and were diagnosed histologically based on the recent WHO classification guidelines. Molecular markers of glioma, such as the IDH1 genotype (IDH1 mutant or wild type), Ki-67 expression status and 1p/19q status (1p/19q codeleted or maintained), were recorded when possible. Immunohistochemistry (IHC) and direct gene sequencing were conducted by pathologists according to routine methods at Beijing Tiantan Hospital (28, 29). The histopathological examination results were verified independently by at least two pathology experts. Other clinical data were extracted from medical records. Corticosteroids such as dexamethasone and methylprednisolone were administered to patients to decrease tumor-associated edema.

Forty-three healthy volunteers, who were matched with the selected glioma patients in terms of sex and age, were included in the healthy control (HC) group.

### Blood Sampling

Peripheral blood samples were obtained upon admission to the hospital before surgery, and clinical parameters [white blood cells (WBCs), neutrophil, lymphocyte, monocyte, and platelet counts, serum albumin and globulin levels] were part of the standard workup at the Laboratory Diagnosis Center of Beijing Tiantan Hospital. In addition, the preoperative NLR (ratio of the neutrophil count to the lymphocyte count), dNLR [ratio of (the WBC count—the neutrophil count) to the lymphocyte count], PLR (ratio of the platelet count to the lymphocyte count), AGR (ratio of the albumin level to the globulin level), and PNI [the albumin level (g/L) + the total lymphocyte count × 5] were calculated.

To assess the temporal dynamics of serum sPD-L1 in glioma patients, we performed monitoring every 3 days postoperatively. Collectively, we obtained postoperative samples as follows: 44 samples at 48 h, 34 samples on day 5, 27 samples on day 8, 9 samples on day 11, 5 samples on day 14 and 4 samples on day 20.

Serum was obtained from each subject during routine venipuncture. To remove blood cells, the serum tubes were centrifuged at 2,500×g for 10 min at room temperature. The separated serum samples were immediately stored at −80°C until analysis. Repeated cycles of freezing and thawing of the samples were avoided.

### CSF Sampling

Thirty-one matched CSF samples from the glioma cohort and seven from the meningioma cohort were collected via lumbar puncture on days 3–6 after resection. The initial 2 mL of CSF was used for protein level measurements and cytology examination. CSF supernatants were transferred to cryotubes and stored at −80°C.

### Measurement of sPD-L1

Both serum and CSF sPD-L1 were examined using a specific enzyme-linked immunosorbent assay (ELISA; PDCD1LG1 ELISA kit, USCN Life Science, Wuhan, China) according to the manufacturer's instructions. In brief, samples to be tested were added to the wells of microtiter plates and incubated for 1 h. Then, the biotinylated antibody was incubated for 1 h, followed by incubation with the avidin-peroxidase conjugate for 30 min. Finally, the substrate TMB was incubated for 15 min, after which the reaction was terminated using H_2_SO_4_. The OD was measured at 450 nm. All steps were run at 37°C. Each sample was tested in duplicate. The detection limit of the ELISA kit was 0.056 ng/mL.

### Statistical Analysis

Continuous variables are presented as the means ± standard deviation (SD) or medians and the minimum-maximum range. Comparisons among different cohorts were performed using analysis of variance (ANOVA) with the Kruskal-Wallis test for nonnormally distributed variables (The *post-hoc* Bonferroni test was used for multiple comparisons). The differences between the two groups were calculated using the *t*-test or Mann-Whitney *U*-test according to the normality of the data. Categorical variables were analyzed and compared between two groups using the chi-squared test or Fisher's exact test. The association of the sPD-L1 with glioma and glioma grade was analyzed using multivariate logistic regression. For Pearson's correlation analysis, sPD-L1 data were log 10 transformed to obtain a more symmetric data distribution. For rank correlation analysis, Spearman's correlation coefficient (rho) was used. A receiver operating characteristic (ROC) curve was generated for each marker. The areas under the curves (AUCs) were assessed to evaluate the performance of each marker for predicting gliomas and distinguishing high- and low-grade gliomas. The dynamics of sPD-L1 in the serum were analyzed by the mixed-model approach. Statistical analysis was performed using SPSS (version 24.0, IBM, New York, USA) or GraphPad Prism 8 software (GraphPad Software Inc., San Diego, CA, USA).

## Results

### Study Subjects

Seventy-three patients with glioma and 20 patients with meningioma were prospectively recruited. In addition, 49 healthy volunteers were included as the control (HC) group. The clinical and demographic features and hematological parameters of the study subjects are listed in Table 1.

**Table 1:** Baseline epidemiological and hematological markers of study populations.

	**Glioma**	**Meningioma**	**HC**	***p***
*n*	73	20	49	
Age (years)	39.88 ± 16.66^§^	53.30 ± 15.73^#^	36.82 ± 10.78	<0.001*
**Sex**				
Male	46 (63.01%)	15 (75%)	31 (63.27%)	0.587
Female	27 (36.99%)	5 (25%)	18 (36.73%)	
sPD-L1 (ng/mL)	0.5594 (0–1.4235)	0.0688 (0.0454–1.4117)	0.1107 (0–0.5908)	<0.001*
**Hematological markers**				
WBC (10^9^/L)	6.69 ± 1.76^#^	6.17 ± 1.67	5.51 ± 1.45	0.001*
Neutrophils (10^9^/L)	4.25 ± 1.47^#^	4.08 ± 1.55^#^	3.17 ± 1.02	<0.001*
Lymphocytes (10^9^/L)	1.92 ± 0.78	1.63 ± 0.59	1.84 ± 0.49	0.234
Monocytes (10^9^/L)	0.38 ± 0.13^§#^	0.32 ± 0.08	0.31 ± 0.09	0.003*
Platelets (10^9^/L)	242.49 ± 62.23^§^	198.80 ± 55.84	222.75 ± 49.97	0.011*
Albumin (g/L)	45.32 ± 3.03^§^	42.78 ± 5.31^#^	45.92 ± 2.36	0.019*
NLR	2.50 ± 1.22^#^	2.93 ± 1.71^#^	1.78 ± 0.55	<0.001*
dNLR	1.28 ± 0.11	1.31 ± 0.11	1.27 ± 0.15	0.688
PLR	141.93 ± 63.11	138.58 ± 66.56	131.96 ± 47.43	0.656
PNI	54.93 ± 5.02^§^	50.94 ± 6.90^#^	55.12 ± 3.49	0.027*
AGR	1.70 ± 0.25	1.61 ± 0.23	1.70 ± 0.23	0.278
**Tumor characteristics**				
Size (cm^3^)	80.00 (1.20–439.28)	43.57 (3.75–741.66)	Na	0.055
Ki-67 (%)	10.0 (1.0–80.0)	5.5 (1.0–30.0)	Na	0.018*
**WHO grade**				
I–II	39 (53.43%)	19 (95%)	Na	<0.001*
III–IV	34 (46.57%)	1 (5%)	Na	
**Steroid therapy**^**a**^				
no	6 (8.2%)	2 (10%)	Na	0.258
≤ 3 days	16 (21.9%)	4 (20%)	Na	
3–7 days	33 (45.2%)	5 (25%)	Na	
≥7 days	18 (24.7%)	9 (45%)	Na	

Data are shown as the means ± SD, medians (range) or number (%).

An asterisk (*) indicates a significant difference.

# P < 0.05 vs. HC.

§ P < 0.05 vs. meningioma.

a At a dose of 10 mg dexamethasone or 40 mg methylprednisolone per day.

*WBC, white blood cell count; NLR, neutrophil-to-lymphocyte ratio; dNLR, derived NLR; PLR, platelet-to-lymphocyte ratio; PNI, prognostic nutritional index; AGR, albumin-to-globulin ratio; Na, not applicable*.

**Figure 1:**
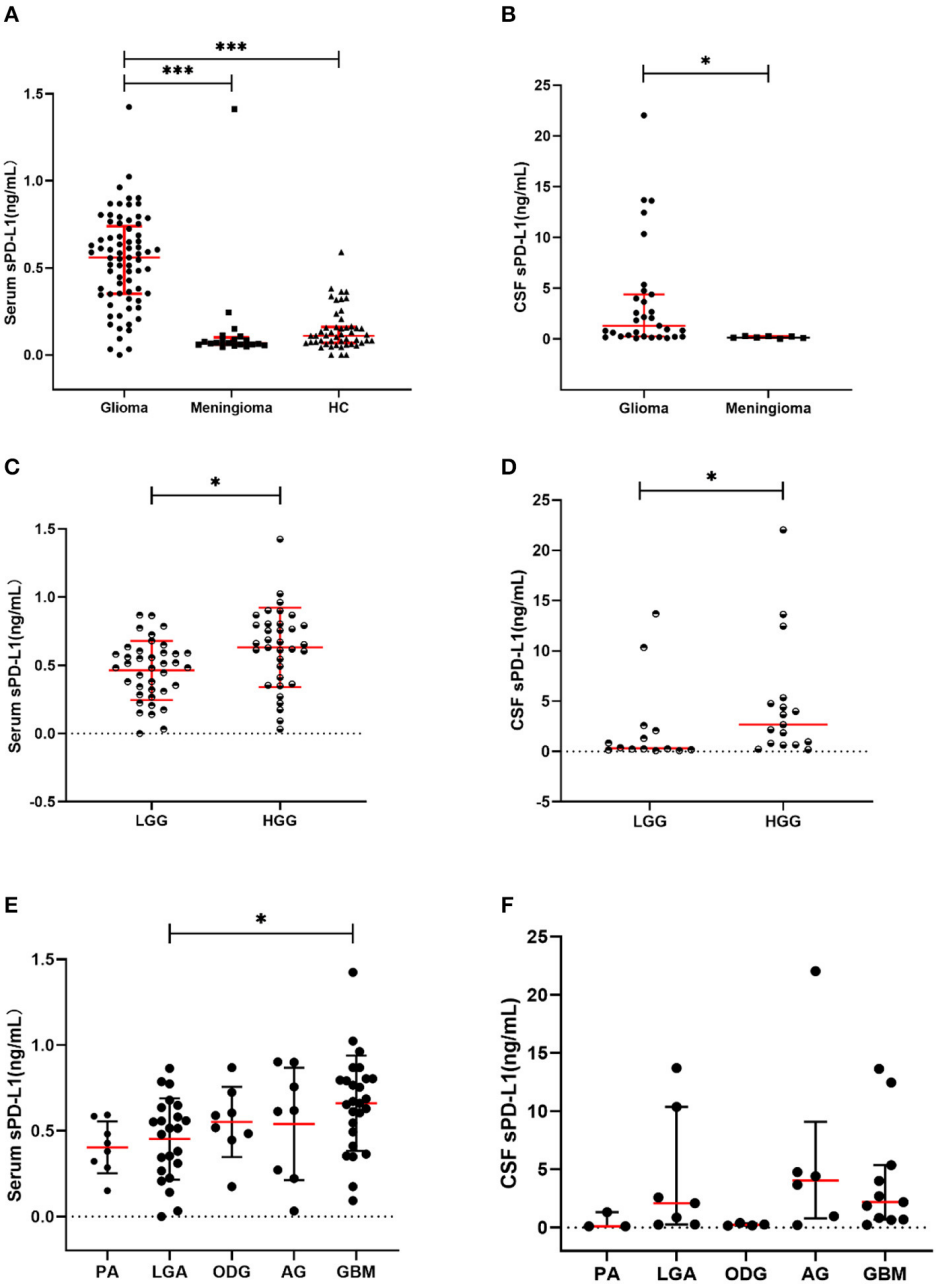
Soluble PD-L1 measurements in study subjects. **(A)** sPD-L1 overexpression in the serum of patients with glioma compared with that of meningioma patients and HCs. **(B)** sPD-L1 levels in the CSF of patients with glioma or meningioma. **(C)** sPD-L1 measurements in the serum of patients with high- or low-grade glioma. **(D)** sPD-L1 measurements in the CSF of patients with high- or low-grade glioma. **(E)** sPD-L1 measurements in the serum of patients with different pathological types of glioma. **(F)** sPD-L1 measurements in the CSF of patients with different pathological types of glioma. For simplicity, only significant differences are shown. The red horizontal lines within the data signify the medians. Statistical significance was defined as *p < 0.05 or ***p < 0.001.

The circulatory sPD-L1 protein levels exhibited the highest concentrations in the glioma cohort (median: 0.5594 ng/mL, range: 0–1.4235 ng/mL) compared with that in the meningioma (0.0688, 0.0454–1.4117; *p* < 0.001) and HC cohorts (0.1107, 0–0.5908; *p* < 0.001) (Table 1 and Figure 1A).

As shown in Table 1 and Table S1, patient age was higher in the meningioma cohort than in the glioma (*p* = 0.001) and HC cohorts (*p* < 0.001). However, when the correlation of serum PD-L1 levels with age was analyzed according to the different cohorts, we did not find any significant differences (Table 2).

**Table 2:** Correlations between varied features and serum sPD-L1 concentrations in different cohorts.

	**Glioma**		**Meningioma**		**HC**	
	**Correlation**	***p***	**Correlation**	***p***	**Correlation**	***p***
Age	0.217	0.066	0.083	0.727	0.271	0.060
WBC	0.010	0.930	−0.180	0.448	0.086	0.555
NEU	0.050	0.675	−0.196	0.409	0.064	0.664
MONO	0.188	0.111	−0.365	0.114	0.299	0.037*
ALB	−0.055	0.647	−0.172	0.468	−0.192	0.186
PLT	−0.058	0.628	0.128	0.592	0.006	0.965
NLR	0.093	0.432	−0.294	0.208	0.039	0.793
PNI	−0.105	0.376	−0.025	0.917	−0.135	0.354
Ki-67	0.246	0.036*	−0.035	0.884	Na	Na
WHO grade	0.387	0.001*	−0.017	0.942	Na	Na

The relationships were assessed by Pearson's or Spearman's rank correlation.

An asterisk (*) indicates a significant difference.

*WBC, white blood cell count; NEU, neutrophils; MONO, monocytes; ALB, albumin; PLT, platelets; NLR, neutrophil-to-lymphocyte ratio; PNI, prognostic nutritional index; Na, not applicable*.

#### Relationship of Serum sPD-L1 to Inflammatory Markers

First, we assessed the differences in peripheral blood markers among the study cohorts. Generally, Table 1 indicates that significant differences were present for WBCs, neutrophils, monocytes, platelets, albumin, the NLR and the PNI.

By performing a *post-hoc* test, we observed significantly higher levels of WBCs, neutrophils, monocytes, and the NLR in the glioma patients than in the HCs (Table S1). Additionally, decreased levels of albumin and the PNI were present in the glioma group compare with that in HCs, although the differences did not reach significance. The levels of monocytes and platelets were increased in the glioma patients compared with meningioma patients (Table S1). Nevertheless, no significant differences were observed for lymphocytes, the dNLR, PLR, or the AGR (Table 1).

When the contributions to serum sPD-L1 levels were further assessed, the inflammatory markers failed to yield any significant associations with serum sPD-L1 in the glioma and meningioma cohorts. Notably, the monocyte count was found to be significantly associated with the sPD-L1 level in the peripheral blood, although the degree was weak (*r* = 0.299, *p* = 0.037) (Table 2).

To determine the association of sPD-L1 with glioma, the parameters that were notably different among the cohorts were further included into a multivariate logistic regression model. As indicated in Table S2, a significantly independent correlation was identified between sPD-L1 and glioma (OR: 1.085, *p* < 0.001). Nevertheless, the other variables (age, WBC, neutrophils, monocytes, platelets, albumin, NLR, and PNI) were not significantly associated with glioma (Table S2).

#### Relationship of Serum sPD-L1 With Brain Tumor Features

As shown in Table 1, the median Ki-67 expression was discernably higher in the glioma cohorts (median: 10.0%, range: 1.0–80.0%) than in the meningioma cohorts (median: 5.5%, range: 1.0–30.0%; *p* = 0.018). Low-grade tumors were more frequently observed in the meningioma cohort vs. the glioma cohort (*p* < 0.001). However, the tumor size was not significantly different (Table 1).

Subsequently, we observed a positive relationship between Ki-67 and sPD-L1 in the glioma patients (*r* = 0.246, *p* = 0.036), and Spearman's rank correlation was markedly positive between the WHO grade and sPD-L1 level (*r* = 0.387, *p* = 0.001). In contrast, neither the Ki-67 index nor the WHO grade was significantly associated with serum sPD-L1 levels in meningioma (Table 2).

#### Association of Serum sPD-L1 With Glioma Grade

According to the WHO criteria (30), grade I–II gliomas were referred to as low-grade gliomas (LGGs), and grade III–IV gliomas were referred to as high-grade gliomas (HGGs). In our cohort, 39 patients were diagnosed with LGG, and 34 were diagnosed with HGG.

In the comparison between LGG and HGG patients, both the Ki-67 index and serum sPD-L1 levels were elevated in HGG patients vs. LGG patients (*p* < 0.001 and *p* = 0.006, respectively). Moreover, the age at the time of diagnosis was younger in LGG patients (*p* = 0.004, Table 3). Regarding the hematological parameters, the levels of neutrophils, monocytes, NLR, dNLR, and PLR were markedly upregulated in HGG patients, whereas notably increased levels of lymphocytes, PNI and AGR were present in LGG patients (Table 3).

**Table 3:** Demographic and hematological parameters between low-grade gliomas and high-grade gliomas.

	**LGGs**	**HGGs**	***P***
*n*	39	34	
Age (year)	34.72 ± 16.66	45.79 ± 14.78	0.004*
Sex (M/F)	26/13	20/14	0.489
Tumor size (cm^3^)	76.95 (1.20–270.0)	98.00 (18.02–439.28)	0.075
Ki-67 (%)	5.0 (1.0–15.0)	35.0 (10.0–80.0)	<0.001*
sPD-L1 (ng/mL)	0.4623 ± 0.2166	0.6316 ± 0.2899	0.006*
**Hematological markers**			
WBC (10^9^/L)	6.34 ± 1.57	7.09 ± 1.9	0.070
Neutrophils (10^9^/L)	3.76 ± 1.2	4.81 ± 1.58	0.002*
Lymphocytes (10^9^/L)	2.09 ± 0.87	1.73 ± 0.6	0.046*
Monocytes (10^9^/L)	0.35 ± 0.11	0.42 ± 0.15	0.015*
Platelets (10^9^/L)	233.44 ± 63.17	252.88 ± 60.4	0.185
Albumin (g/L)	45.95 ± 3.12	44.62 ± 2.82	0.062
NLR	2.00 ± 0.79	3.09 ± 1.37	<0.001*
dNLR	1.24 ± 0.08	1.33 ± 0.12	<0.001*
PLR	122.39 ± 46.45	164.34 ± 72.37	0.005*
PNI	56.39 ± 5.48	53.26 ± 3.89	0.007*
AGR	1.77 ± 0.24	1.63 ± 0.24	0.012*

An asterisk (*) indicates a significant difference.

*NLR, neutrophil-to-lymphocyte ratio; dNLR, derived NLR; PLR, platelet-to-lymphocyte ratio; PNI, prognostic nutritional index; AGR, albumin-to-globulin ratio*.

However, similar to the results shown above, we did not find any significant correlations between blood-based markers and serum sPD-L1 with respect to the glioma grade (Table S3).

Next, the predictive value of these variables in high-grade glioma was assessed using a multivariate stepwise logistic regression analysis. The analysis revealed that sPD-L1 (OR: 1.030, *p* = 0.013) and NLR (OR: 2.850, *p* = 0.001) were independent factors for the prediction of HGGs, while the other parameters (age, neutrophils, lymphocytes, monocytes, dNLR, PLR, PNI, and AGR) were not independent predictors (Table S4).

Collectively, these results suggest that the occurrence of aggressive neoplasms in the brain may explain the elevated sPD-L1 levels in the serum.

### Dynamics of sPD-L1 in the Serum of Glioma Patients

**Figure 2:**
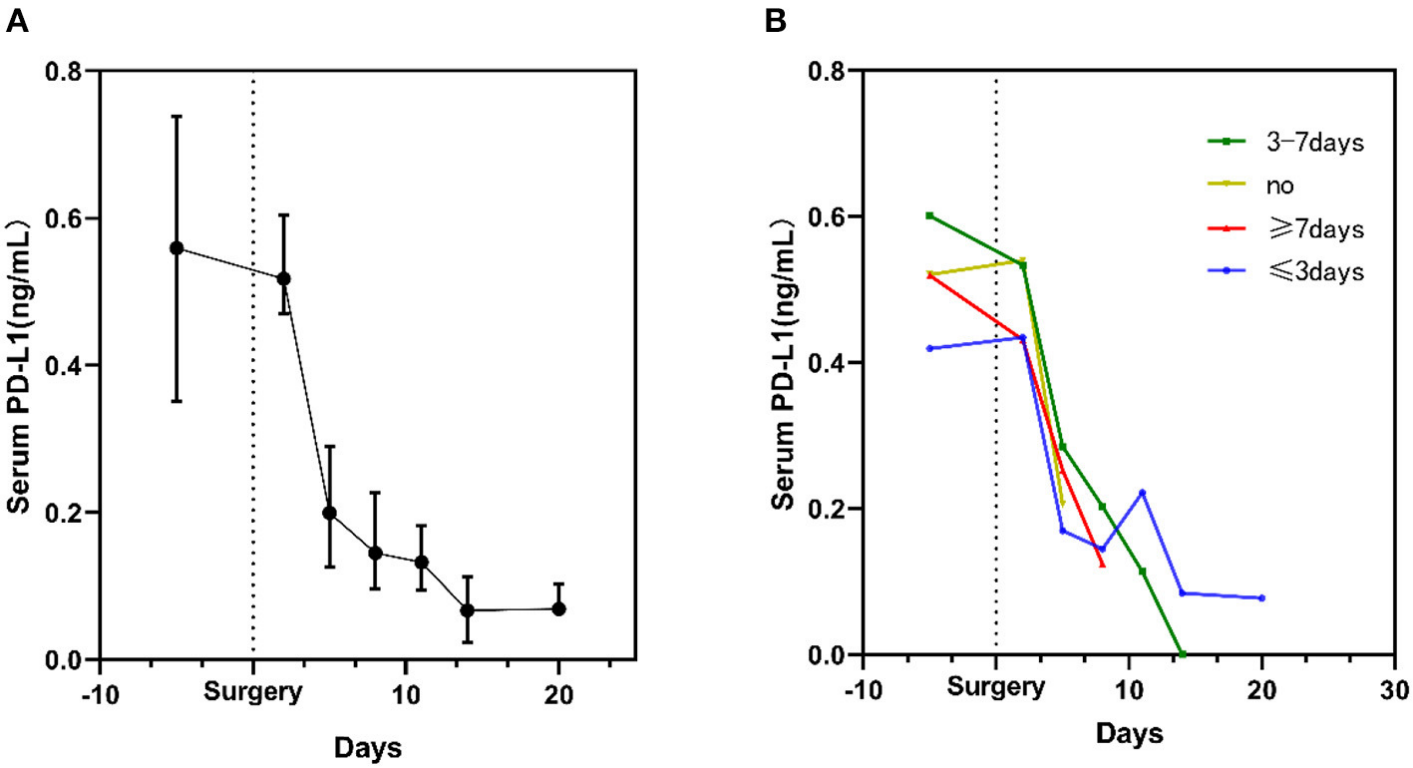
Kinetic changes in serum sPD-L1 levels in glioma patients during the perioperative period. **(A)** Kinetic changes in all glioma patients. Dots display the medians, and whiskers represent the interquartile ranges. **(B)** Serum sPD-L1 levels in patients stratified by the time of exposure to steroids. The plots display the medians.

After 20 days of post-surgery surveillance, a decline in the sPD-L1 level was noted during the postoperative period. Notably, the level of sPD-L1 was dramatically decreased on the 5th day after resection. In addition, the lowest level was observed on the 14th day after surgery (Figure 2A).

In total, 67 glioma patients received concomitant antiedematous therapy during the perioperative period (Table 1). However, as depicted in Figure 2B, the sPD-L1 levels remained decreased after surgery in the six patients who did not receive steroid treatment (yellow curve in Figure 2B). A similar phenomenon was observed in other patients who were administered steroids for different periods of time (Figure 2B).

### CSF sPD-L1 in Brain Tumor Patients

To complete the analysis of sPD-L1 in brain tumor patients, we further evaluated sPD-L1 expression in the CSF. Globally, 38 matched CSF samples including 31 from glioma patients and seven from meningioma patients were obtained from brain tumor patients after resection. The characteristics of the CSF samples are shown in Table 4.

**Table 4:** CSF features of brain tumors after resection.

	**Glioma**	**Meningioma**	***p***
*N*	31	7	–
CSF sPD-L1 (ng/mL)	1.3202 (0.0925–22.0392)	0.1538 (0.0475–0.3157)	0.004*
**Characteristics**			
Protein (mg/dL)	106.10 (25.40–3,213.00)	81.00 (28.30–134.20)	0.179
Nucleated cells (cells/mm^3^)	5,266 (42–156,468)	6,747 (2–39,158)	0.658
WBC (cells/mm^3^)	847 (7–10,376)	58 (2–3,311)	0.049*
PMN (cells/mm^3^)	82.0 (0–96.4)	51.7 (0–90.6)	0.125
MN (cells/mm^3^)	16.6 (0–80.5)	14.9 (0–48.7)	0.632

An asterisk (*) indicates a significant difference.

*PMN, polymorphonuclear cells; MN, mononuclear cells; WBC, white blood cells*.

Increased levels of sPD-L1 were found in the CSF of glioma patients (1.3202 ng/mL, 0.0925–22.0392 ng/mL; *p* = 0.003) compared with that of meningioma patients (0.1538 ng/mL, 0.0475–0.3157 ng/mL) (Table 4 and Figure 1B).

As shown in Table 4, a difference in the WBC count was observable between the glioma and meningioma CSF samples. In addition, PD-L1 expression appeared to be notably associated with the WBC count in the glioma CSF samples (*r* = 0.577, *p* = 0.001), but this relationship was not significant in the meningioma CSF samples (*r* = 0.162, *p* > 0.05).

Similar to the findings in the serum, elevated levels of sPD-L1 were observed in the CSF of patients with more advanced grade gliomas (*p* = 0.029) (Table S5 and Figure 1D). Nevertheless, the other CSF parameters failed to show any significant variations between LGG patients and HGG patients (Table S5).

### Association Between Serum and CSF PD-L1 Expression

The glioma cohort consisted of patients with different histopathological types of disease, including pilocytic astrocytoma (PA), low-grade astrocytoma (LAG), oligodendroglioma (ODG), anaplastic glioma (AG), and glioblastoma (GBM). The sPD-L1 levels in the serum and CSF for each disease type are shown in Table 5.

**Table 5:** Serum and CSF sPD-L1 levels in gliomas stratified by disease features.

	**Serum**			**CSF**		
	***n***	**sPD-L1**	***p***	***n***	**sPD-L1**	***p***
	73	0.5594 (0–1.4235)	–	31	1.3202 (0.0925–22.0392)	–
**WHO** **grade**						
LGG	39	0.4623 ± 0.2166	0.006*	14	0.325 (0.093–13.701)	0.029*
HGG	34	0.6316 ± 0.2899		17	2.676 (0.199–22.039)	
**Pathology**						
PA	8	0.4029 ± 0.1512	0.036*	3	0.099 (0.092–1.32)	0.032*
LGA	23	0.4521 ± 0.2370		7	1.470 (0.14–13.701)	
AG	8	0.5393 ± 0.3273		6	4.034 (0.20–22.04)	
ODG	8	0.5509 ± 0.2047		4	0.259 (0.18–0.39)	
GBM	26	0.6600 ± 0.2782		11	2.171 (0.23–13.63)	
**Tumor** **size**						
≤80 cm^3a^	37	0.5710 ± 0.2448	0.333	14	0.454 (0.092–13.634)	0.010*
>80 cm^3^	36	0.5104 ± 0.2855		17	2.581 (0.199–22.039)	
Ki-67						
≤10%^a^	37	0.4618 ± 0.2263	0.009*	14	0.325 (0.093–13.701)	0.029*
>10%	36	0.6227 ± 0.2809		17	2.676 (0.199–22.039)	
**IDH-1** **type**						
Mutant	37	0.5509 ± 0.2973	0.595	18	0.823 (0.18–22.039)	0.042*
Wild type	22	0.5918 ± 0.2595		8	3.172 (0.15–10.36)	
**1p19q** **status**						
Codeleted	12	0.5349 ± 0.2341	0.672	7	0.259 (0.14–4.77)	0.017*
Maintained	47	0.5740 ± 0.2949		19	2.676 (0.23–22.039)	

An asterisk (*) indicates a significant difference.

a The median value in the glioma cohort.

*PA, pilocytic astrocytoma; LGA, low-grade astrocytoma; ODG, oligodendroglioma; AG, anaplastic glioma; GBM, glioblastoma; IDH, isocitrate dehydrogenase*.

#### Associations of sPD-L1 Levels With Disease Features in Glioma

The differences among different pathological types were notable when serum sPD-L1 was used as a marker (*p* = 0.036). As indicated in Figure 1E, the serum sPD-L1 concentration was markedly higher in GBM than in LGA (*p* = 0.043). Similar to the PD-L1 expression pattern in the serum, the CSF PD-L1 level differed according to histological type (*p* = 0.032), but no significant differences were found between any two types (Figure 1F).

When the sPD-L1 level difference was analyzed according to tumor size, the difference in CSF sPD-L1 was significant, but the difference in serum sPD-L1 was not, suggesting that the effect of tumor size on CSF PD-L1 measurements was stronger than that on serum sPD-L1 measurements. In addition, samples with high Ki-67 expression had significantly higher sPD-L1 levels in both the serum and CSF than samples with low Ki-67 expression.

Overall, a higher sPD-L1 level might reflect more aggressive histopathological features in glioma.

#### Associations of sPD-L1 Levels With Molecular Characteristics

As shown in Table 5, we did not find any differences in serum sPD-L1 related to the IDH1 genotype or 1p/19q status. Next, we explored the associations between molecular subtypes and CSF sPD-L1. The glioma patients with mutated IDH-1 (*p* = 0.042) or 1p/19q codeletion (*p* = 0.017) were found to have relatively low levels of CSF sPD-L1.

#### Correlation Between Serum and CSF Measurements

**Figure 3:**
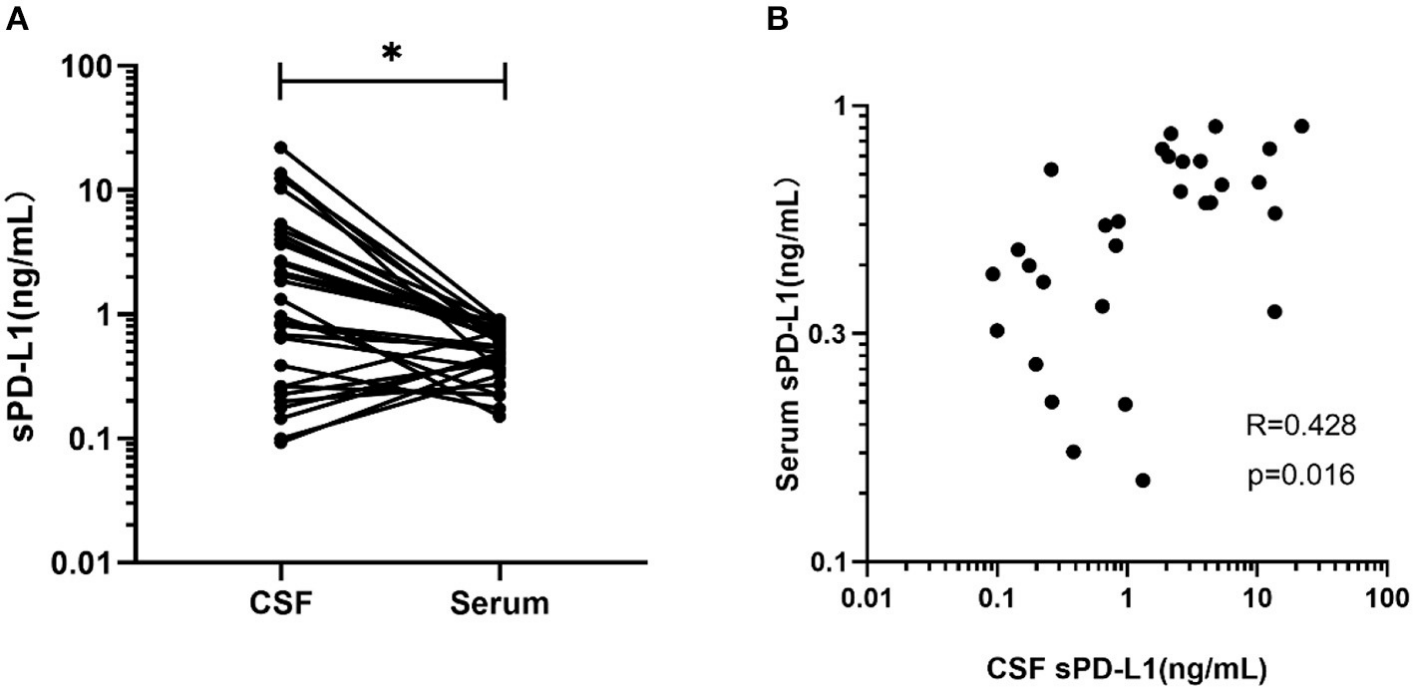
Comparison and correlation between sPD-L1 in the serum and matched CSF of glioma patients. **(A)** sPD-L1 measurements in CSF and matched serum samples collected from 31 patients. **(B)** Correlation between serum and CSF sPD-L1 levels in individual patients with gliomas (*n* = 31). Pearson's correlation coefficients (R) and *P*-values are shown. Statistical significance was defined as *p < 0.05.

Thirty-one glioma patients had both their serum and CSF sPD-L1 levels measured. As indicated in Figure 3A, the median CSF sPD-L1 concentration was 1.32 (0.09–22.04) ng/mL, which was significantly higher than the level in the serum (0.56 ng/mL, 0–1.42 ng/mL; *p* = 0.002).

The level in the serum appeared to positively correlate with the level in the CSF of the glioma patients (*r* = 0.428, *p* = 0.016, Figure 3B).

### Evaluation of the Diagnostic Efficacy of Inflammatory Markers and sPD-L1 in Gliomas

**Figure 4:**
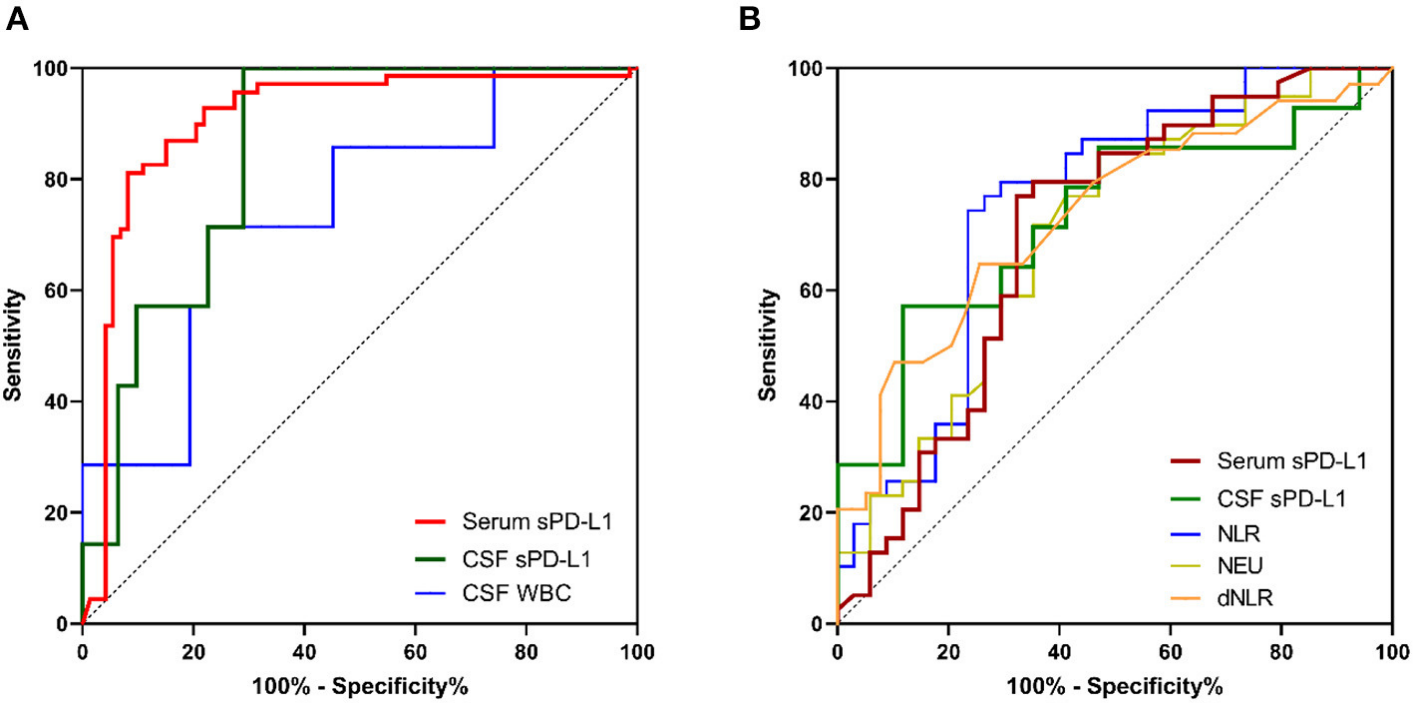
The diagnostic value of inflammatory markers and sPD-L1 in gliomas. **(A)** ROC curves for predicting gliomas. **(B)** ROC curves for distinguishing high-grade gliomas from low-grade gliomas. For simplicity, only the ROC curves for which an AUC ≥ 0.70 are shown.

As indicated in Table 6, AUCs generated by ROC analysis showed the diagnostic efficacy of different inflammatory markers in glioma patients. When blood-based biomarkers in glioma patients were compared with the corresponding markers in healthy volunteers and meningioma patients, we observed that serum sPD-L1 had the best value for the diagnosis of glioma [0.906 (0.850–0.962), Figure 4A]. In addition, the efficacy of CSF sPD-L1 was demonstrated to be significant in distinguishing glioma from meningioma (Figure 4A).

**Table 6:** Diagnostic value of inflammatory markers in gliomas.

**Parameters**	**AUC (95% CI)**	
	**Glioma vs. other**	**HGG vs. LGG**
Serum sPD-L1	0.906 (0.850–0.962)*	0.702 (0.577–0.827)*
CSF sPD-L1	0.853 (0.728–0.977)*	0.731 (0.545–0.917)*
WBC in the CSF	0.742 (0.538–0.946)*	0.571 (0.366–0.777)
NLR	0.628 (0.535–0.720)*	0.752 (0.636–0.868)*
dNLR	0.514 (0.418–0.609)	0.733 (0.616–0.849)*
NEU	0.672 (0.582–0.761)*	0.700 (0.578–0.821)*
WBC	0.667 (0.578–0.755)*	0.619 (0.488–0.750)
MONO	0.657 (0.567–0.748)*	0.654 (0.528–0.779)*
PLT	0.605 (0.511–0.698)*	0.602 (0.471–0.734)
LY	0.517 (0.421–0.612)	0.647 (0.518–0.777)*
ALB	0.494 (0.398–0.590)	0.656 (0.529–0.784)*
PLR	0.517 (0.421–0.613)	0.682 (0.556–0.804)*
PNI	0.491 (0.395–0.586)	0.689 (0.566–0.812)*
AGR	0.541 (0.446–0.636)	0.676 (0.552–0.800)*

Other includes healthy controls and patients with meningioma.

An asterisk (*) indicates a significant difference.

*AUC, area under the curve; WBC, white blood cell count; LY, lymphocytes; dNLR, derived NLR; NEU, neutrophils; MONO, monocytes; ALB, albumin; PLT, platelets; NLR, neutrophil-to-lymphocyte ratio; PLR, platelet-to-lymphocyte ratio; AGR, albumin-to-globulin ratio; PNI, prognostic nutritional index*.

Similarly, we investigated the diagnostic value of each marker for predicting HGG. As shown in Table 6 and Figure 4B, both serum sPD-L1 and CSF sPD-L1 showed notable predictive value for HGG. Furthermore, a higher accuracy in distinguishing HGG from LGG was achieved with CSF sPD-L1 levels than with serum sPD-L1 levels [0.731 (0.545–0.917) vs. 0.702 (0.577–0.827), respectively, Figure 4B].

## Discussion

At present, the difficulties associated with performing biopsies hamper glioma diagnosis and therapeutic intervention, highlighting the need to discover non-invasive or minimally invasive biomarkers for early diagnosis and correct stratification (31). sPD-L1 can be detected in biofluids, providing a novel diagnostic test for a variety of tumors (32–34). In our previous study, elevated sPD-L1 levels in the serum were observed in preoperative patients with HGG. In this study, we endeavored to collect laboratory parameters and tumor characteristics to investigate the clinical significance of sPD-L1 in gliomas. Thus, we recruited patients with benign tumors as controls to test the potential role of sPD-L1 in malignant brain tumors.

Consistent with our previous results, overexpression of circulating sPD-L1 occurred in glioma patients (median: 0.5594 ng/mL, range: 0–1.4235 ng/mL), while the concentrations were notably lower in HCs (0.1107, 0–0.5908, *p* < 0.001) and meningioma patients (0.0688, 0.0454–1.4117, *p* < 0.001) (Figure 1A). Likewise, the enhanced level of serum sPD-L1 was reproducibly present in HGG (Table 3).

It has been documented that inflammation is involved in the pathogenesis and promotion of cancer (35). Therefore, blood components, such as WBCs, neutrophils, lymphocytes, monocytes, platelets and albumin, have been widely used for detecting the host inflammatory response in cancers (6, 36). Furthermore, inflammation-based scores derived from the abovementioned parameters, such as the NLR, PLR, dNLR, AGR, and PNI, have been demonstrated to be positively associated with glioma grade and negative outcomes (7–9, 37). Notably, these peripheral blood biomarkers have been recently adopted for predicting the outcomes of patients treated with checkpoint inhibitors (38–40). In addition, accumulating findings indicate that an elevated sPD-L1 level is a kind of sign of systematic inflammation provoked by neoplasms (21, 41). Given this aspect, we first investigated whether there are associations between serum sPD-L1 and inflammatory markers in preoperative gliomas. In agreement with the above results, we observed increased levels of WBCs, neutrophils, monocytes and the NLR in glioma patients, and relatively low levels of albumin and the PNI were also expressed in the glioma patients (Table 1). In addition, the inflammation scores such as NLR, dNLR, and PLR were markedly upregulated in high-grade gliomas, which were consistent with the results of previous studies (6, 42, 43). Regardless, the serum sPD-L1 levels failed to yield any significant associations with systemic inflammatory markers in the glioma patients (Tables 2, 3).

The multivariate analysis revealed that sPD-L1 is independently associated with glioma after adjusting for age and the mentioned hematological markers (Table S2). For glioma diagnosis, the AUC obtained from the ROC curve was 0.906 (0.850–0.962) for serum sPD-L1, which was higher than that for the NLR [0.628 (0.535–0.720)], WBCs [0.667 (0.578–0.755)], neutrophils [0.672 (0.582–0.761)], and monocytes [0.657 (0.567–0.748)]. Accordingly, serum sPD-L1 showed better diagnostic performance than the abovementioned inflammatory markers. When the diagnostic power was further evaluated to distinguish HGGs from LGGs, the performance of circulatory sPD-L1 levels was significant (AUC: 0.702). Even after multivariate analysis, sPD-L1 retained its power to independently predict HGGs (OR: 1.030; 95% CI: 1.006–1.054, *p* = 0.013). Collectively, this observation has highlighted the potential role of sPD-L1 in glioma diagnosis and stratification.

Next, we attempted to uncover the perioperative dynamics of circulating sPD-L1 levels in glioma patients. The expression of sPD-L1 in the serum was prospectively evaluated after craniotomy for up to 20 days. In this context, the sPD-L1 levels in the circulation were observed to consistently decrease after tumor removal, which persisted regardless of the use of steroids (Figure 2B). The serum levels were markedly decreased on the 5th day after surgical resection. Some published studies assert that the sPD-L1 level after treatment may reflect the residual tumor burden (44, 45). Since all the glioma patients underwent maximal safe surgical resection, we speculated that the removal of the tumor might partly explain the decline in serum sPD-L1. However, it was reported that the low level of sPD-L1 indicated inflammation suppression (46). Taking into account the fact that most patients had received steroid therapy for edema (47), and the steroids are known to cause systemic immunosuppression (48), the decrease in the circulating sPD-L1 level might be affected by the use of steroids. Nevertheless, the correlation between serum sPD-L1 and steroids remains ambiguous. sPD-L1 is a dynamic marker, varying across time points; therefore, long-term studies are needed to examine the potential clinical relevance in the future.

Herein, an elevated serum sPD-L1 level was present in patients with relatively advanced brain tumors, as expected (Tables 1, 3 and Figure 1). Combined with our unpublished results demonstrating that an elevated sPD-L1 level could predict reduced progression-free survival in patients with gliomas, we hypothesized that sPD-L1 is involved in the aggressive biological activities of tumors. Notably in this context, patients with high Ki-67 expression had significantly increased circulating sPD-L1 levels (Table 5). Ki-67 has been validated as a marker of proliferation in the initial phase of adult neurogenesis and is used clinically to assess tumor cell proliferative activity in diverse tumor types (49). Although the mechanistic link remains unclear, we suggest that sPD-L1 represents the PD-L1 expression in tumor tissue (25), which is accompanied by suppression of the immune response. In addition, the soluble form of PD-L1 is biologically activated in compromised antitumor immune responses (19). Both scenarios lead to immune tolerance; consequently, neoplastic cells would have no limits to proliferation. Therefore, abnormal expression of sPD-L1 may be an indicator of the occurrence and development of brain malignances. Furthermore, the serum sPD-L1 level could provide a tool for monitoring treatment response.

There are two ways for a brain protein to access the peripheral blood, that is, by CSF flow into the venous blood or by penetrating though the BBB (50). Given this information, we suspected that the high levels of sPD-L1 in the peripheral blood were due to molecules leaking from the CSF. To explore this relationship, we evaluated the expression of CSF sPD-L1 in matched patients. In our case series, we found excessive sPD-L1 expression in the CSF of glioma patients compared with that of meningioma patients (Figure 1B), suggesting that CSF sPD-L1 is released from a large number of tumor cells. Moreover, the expression of sPD-L1 was dramatically increased in the CSF compared with that in matched serum [1.3202 (0.0925–22.0392) vs. 0.5594 (0–1.4235), respectively, *p* = 0.002]. Subsequently, a positive correlation between serum and CSF PD-L1 levels was observed, although the degree of significance was mild (*r* = 0.428).

Another important purpose of this study was to investigate the roles of sPD-L1 in different biofluids for the diagnosis and prediction of gliomas. For this purpose, we evaluated the differences in the sPD-L1 levels in the serum and CSF according to varied clinicopathological features of gliomas. Similar to the expression in the serum, sPD-L1 expression in the CSF was higher in more advanced gliomas than in less advanced gliomas (Table 5). However, regarding tumor size and molecular markers, the differences remained statistically significant in the CSF but not in the serum. An explanation for the results may be that the CSF is in direct contact with the CNS (51), which indicates that CSF sPD-L1 may be more suitable for CNS malignancies than serum sPD-L1 in clinical practice.

Numerous published reports have declared that an IDH mutation or 1p/19q codeletion can affect the pathological behaviors of gliomas (52, 53). These molecular parameters were incorporated into the 2016 WHO classification schema for brain tumors and have paved the way for more precise drug therapies for gliomas (2). Several lines of evidence have demonstrated that mutation of IDH1 indicates enhanced chemosensitivity and is associated with an improved prognosis in glioma patients (54). Additionally, it has been reported that glioma with 1p/19q codeletion is sensitive to alkylating agents and tends to have prolonged survival (55). In this study, the upregulated CSF sPD-L1 levels tended to occur in the glioma patients with wild-type IDH-1 or maintained 1p/19q. This finding might partly explain why the enhanced sPD-L1 levels were related to unfavorable outcomes in the glioma patients in our previous study. On the other hand, the detection of CSF sPD-L1 could be adopted to help make decisions for postsurgical treatments at an early phase.

It is much more preferable to adopt CSF evaluation than peripheral blood evaluation for diagnostic analysis since the CSF directly contacts the CNS. Conversely, the AUC for serum sPD-L1 was markedly higher than that of CSF sPD-L1 for predicting gliomas [0.906 (0.850–0.962) vs. 0.853 (0.728–0.977), respectively] in this context. Moreover, the blood-based biomarkers such as NLR (AUC: 0.752) and dNLR (0.733) showed a better performance than CSF sPD-L1 (0.731) for the differentiation of gliomas. The lack of predictive power of CSF sPD-L1 might be due to the small number of CSF samples; therefore, further studies involving a larger cohort of patients might be needed to investigate the role of sPD-L1 in the CSF.

This study has several limitations. Although we detected upregulated levels of serum sPD-L1 among preoperative patients with glioma and observed a descending trend during the early postsurgical phase, it is not known whether sPD-L1 levels were affected by the concomitant therapy and whether sPD-L1 levels in serum fluctuate during tumor progression or remission. In addition, further investigation is required to determine whether sPD-L1 is robustly correlated with tumor activity, which would validate this soluble protein as a screening tool in future studies.

## Conclusion

Our findings show the significant value of sPD-L1 in the serum and CSF for the diagnosis and discrimination of gliomas, providing the rationale to further study the role of sPD-L1 as a surrogated biomarker for the future clinical management of gliomas. Thus, the detection of sPD-L1 by intravenous or lumbar puncture rather than tumor tissue sampling could impact treatment decisions.

## Data Availability Statement

All datasets generated for this study are included in the article/Supplementary Material.

## Ethics Statement

The studies involving human participants were reviewed and approved by the ethics committee of Beijing Tiantan Hospital. The patients/participants provided their written informed consent to participate in this study.

## Author Contributions

SL and YZ: study concept and design. SL, GZ, and CZ: methodology. SL, CZ, and XM: experiments. SL and BS: analysis and interpretation of data. SL: writing-original draft preparation. YF and XK: writing-review and editing. XK: supervision.

### Conflict of Interest

The authors declare that the research was conducted in the absence of any commercial or financial relationships that could be construed as a potential conflict of interest.
