# Supplementary material for: The Clinical Significance of Soluble Programmed Cell Death-Ligand 1 (sPD-L1) in Patients With Gliomas
Source: Front Oncol. 2020 Jan 23;10:9. doi: 10.3389/fonc.2020.00009 (PMC6989542; doi:10.3389/fonc.2020.00009)
Supplement: Supplementary file 1 [file Table_1.DOCX]

**Supplementary tables**

Table S1 Multiple comparisons for different cohorts

|  | G/M | | G/C | | M/C | |
| --- | --- | --- | --- | --- | --- | --- |
|  | Z | p value | Z | p value | Z | p value |
| Age (year) | -13.42 | 0.001^*^ | 3.060 | 0.502 | 16.48 | <0.001^*^ |
| Sex (M/F) | Na | 0.317 | Na | 0.977 | Na | 0.384 |
| sPD-L1 (ng/mL) | 0.4906 | <0.001^*^ | 0.4487 | <0.001^*^ | -0.0419 | 0.986 |
| WBC (10^9^/L) | 0.516 | 0.430 | 1.173 | 0.001^*^ | 0.657 | 0.291 |
| Neutrophils (10^9^/L) | 0.166 | 0.877 | 1.079 | <0.001^*^ | 0.913 | 0.031^*^ |
| Lymphocytes (10^9^/L) | 0.286 | 0.207 | 0.079 | 0.793 | -0.207 | 0.473 |
| Monocytes (10^9^/L) | 0.067 | 0.050^*^ | 0.021 | 0.006^*^ | 0.031 | >0.999 |
| Platelets (10^9^/L) | 43.69 | 0.008^*^ | 13.74 | 0.399 | -29.95 | 0.125 |
| Albumin (g/L) | 2.554 | 0.006^*^ | -0.589 | 0.589 | -3.143 | 0.001^*^ |
| NLR | -0.426 | 0.297 | 0.728 | 0.002^*^ | 1.154 | 0.001^*^ |
| dNLR | -0.015 | 0.886 | 0.013 | 0.842 | 0.028 | 0.682 |
| PLR | 3.346 | 0.972 | 9.962 | 0.629 | 6.617 | 0.905 |
| PNI | 3.985 | 0.004^*^ | -0.191 | 0.976 | -4.176 | 0.004^*^ |
| AGR | 0.094 | 0.266 | 0.004 | 0.995 | -0.090 | 0.333 |

An asterisk (*) indicates a significant difference.
G: glioma group; M: meningioma group; C: healthy controls; WBC: white blood cell count; NLR: neutrophil-to-lymphocyte ratio; dNLR: derived NLR; PLR: platelet-to-lymphocyte ratio; PNI: prognostic nutritional index; AGR: albumin-to-globulin ratio; Na: not applicable.

Table S2 Multivariate regression of potent markers associated with gliomas

|  | B | OR | 95%CI | p |
| --- | --- | --- | --- | --- |
| Age (year) | 0.006 | 1.006 | 0.959~1.056 | 0.797 |
| sPD-L1 (10^-2^ ng/mL) | 0.081 | 1.085 | 1.051~1.119 | <0.001^*^ |
| WBC (10^9^/L) | -1.509 | 0.221 | 0.003~15.692 | 0.488 |
| Neutrophils (10^9^/L) | 1.233 | 3.431 | 0.038~312.944 | 0.592 |
| Monocytes (10^7^/L) | 0.001 | 1.001 | 0.919~1.091 | 0.979 |
| Platelets (10^9^/L) | -0.001 | 0.999 | 0.988~1.011 | 0.932 |
| Albumin (g/L) | -0.744 | 0.475 | 0.165~1.365 | 0.167 |
| NLR | 2.270 | 9.681 | 0.801~117.037 | 0.074 |
| PNI | 0.711 | 2.037 | 0.721~5.755 | 0.179 |

An asterisk (*) indicates a significant difference.
OR: odds ratio; CI: confidence interval; NLR: neutrophil-to-lymphocyte ratio; PNI: prognostic nutritional index.

Table S3 Correlations between blood-based markers and serum sPD-L1 levels by glioma grade

|  | LGGs | | HGGs | |
| --- | --- | --- | --- | --- |
|  | Correlation | p | Correlation | p |
| Age | 0.118 | 0.475 | 0.133 | 0.454 |
| WBC | 0.092 | 0.576 | 0.064 | 0.720 |
| Neu | 0.107 | 0.518 | 0.050 | 0.778 |
| LY | 0.142 | 0.389 | 0.017 | 0.923 |
| Mono | 0.074 | 0.654 | 0.220 | 0.211 |
| NLR | -0.186 | 0.256 | 0.017 | 0.926 |
| dNLR | 0.004 | 0.981 | 0.143 | 0.419 |
| PLR | -0.039 | 0.816 | -0.176 | 0.318 |
| PNI | 0.041 | 0.803 | -0.009 | 0.962 |
| AGR | -0.292 | 0.072 | -0.075 | 0.672 |

WBC: white blood cell count; NEU: neutrophils; MONO: monocytes; LY: lymphocytes; NLR: neutrophil-to-lymphocyte ratio; dNLR: derived NLR; PLR: platelet-to-lymphocyte ratio; PNI: prognostic nutritional index; AGR: albumin-to-globulin ratio.

Table S4 Multivariate logistic regression of blood-based markers associated with HGG

|  | B | OR | 95%CI | p |
| --- | --- | --- | --- | --- |
| PD-L1(10^-2^ng/mL) | 0.030 | 1.030 | 1.006-1.054 | 0.013^*^ |
| NLR | 1.047 | 2.850 | 1.532-5.302 | 0.001^*^ |

An asterisk (*) indicates a significant difference.
OR: odds ratio; CI: confidence interval; NLR: neutrophil-to-lymphocyte ratio.

Table S5 CSF features by glioma grade

|  | LGGs | HGGs | p |
| --- | --- | --- | --- |
| N | 14 | 17 | - |
| CSF sPD-L1 (ng/mL) | 0.325 (0.093-13.701) | 2.676 (0.199-22.039) | 0.029^*^ |
| Characteristics |  |  |  |
| Protein (mg/dL) | 96.30 (25.40-233.60) | 201.50 (29.50-3,213) | 0.052 |
| Nucleated cells (cells/mm^3^) | 1,709.5 (42-107,959) | 9,435 (81-156,468) | 0.552 |
| WBC (cells/mm^3^) | 833 (7-8,990) | 847 (10-10,376) | 0.500 |
| PMN (cells/mm^3^) | 85.7 (0-96.4) | 81.4 (23.6-95.5) | 0.843 |
| MN (cells/mm^3^) | 12.7 (0-80.5) | 18.6 (4.5-76.4) | 0.634 |

An asterisk (*) indicates a significant difference.
PMN: polymorphonuclear cells; MN: mononuclear cells; WBC: white blood cells.
